# Automated description of the mandible shape by deep learning

**DOI:** 10.1007/s11548-021-02474-2

**Published:** 2021-08-27

**Authors:** Nicolás Vila-Blanco, Paulina Varas-Quintana, Ángela Aneiros-Ardao, Inmaculada Tomás, María J. Carreira

**Affiliations:** 1 Centro Singular de Investigación en Tecnoloxías Intelixentes (CiTIUS) and Departamento de Electrónica e Computación, Universidade de Santiago de Compostela, Santiago de Compostela, Spain; 2 Oral Sciences Research Group, Special Needs Unit Department of Surgery and Medical-Surgical Specialities School of Medicine and Dentistry, Universidade de Santiago de Compostela, Santiago de Compostela, Spain; 3 Instituto de Investigación Sanitaria de Santiago de Compostela (IDIS), Santiago de Compostela, Spain

**Keywords:** Convolutional neural networks, Shape modeling, Mandible morphometrics, Deep learning

## Abstract

****Purpose**:**

The shape of the mandible has been analyzed in a variety of fields, whether to diagnose conditions like osteoporosis or osteomyelitis, in forensics, to estimate biological information such as age, gender, and race or in orthognathic surgery. Although the methods employed produce encouraging results, most rely on the dry bone analyses or complex imaging techniques that, ultimately, hamper sample collection and, as a consequence, the development of large-scale studies. Thus, we proposed an objective, repeatable, and fully automatic approach to provide a quantitative description of the mandible in orthopantomographies (OPGs).

****Methods**:**

We proposed the use of a deep convolutional neural network (CNN) to localize a set of landmarks of the mandible contour automatically from OPGs. Furthermore, we detailed four different descriptors for the mandible shape to be used for a variety of purposes. This includes a set of linear distances and angles calculated from eight anatomical landmarks of the mandible, the centroid size, the shape variations from the mean shape, and a group of shape parameters extracted with a point distribution model.

****Results**:**

The fully automatic digitization of the mandible contour was very accurate, with a mean point to the curve error of 0.21 mm and a standard deviation comparable to that of a trained expert. The combination of the CNN and the four shape descriptors was validated in the well-known problems of forensic sex and age estimation, obtaining 87.8% of accuracy and a mean absolute error of 1.57 years, respectively.

****Conclusion**:**

The methodology proposed, including the shape model, can be valuable in any field that requires a quantitative description of the mandible shape and a visual representation of its changes such as clinical practice, surgery management, dental research, or legal medicine.

**Supplementary Information:**

The online version contains supplementary material available at 10.1007/s11548-021-02474-2.

## Introduction

The mandible is the strongest, largest, and only movable facial bone [1]. It enables speech and mastication and hosts the lower teeth. As a consequence, mandible disorders have a significant effect on both appearance and quality of life. Furthermore, examinations of the mandible’s form can be employed in the diagnosis of several conditions [2–7].

Dentistry, orthodontics, and forensics have probably been the fields where the mandible bone has been studied the most. Regarding the latter, many works reported a strong relationship between mandibular bone features, such as morphometry and appearance, and biological variables as sex or age. Gender dimorphism has been assessed through a set of distances between anatomical landmarks [8–10] or the analysis of the mandibular shape [11]. It is worth noting that the sex estimation models reported a high accuracy for adult subjects, as the gender dimorphism is higher than in subadults [12]. The mandible evolution with age has been studied to a lesser extent, with the opposite finding, that is, the mandible changes in older people are quite limited and mainly related to tooth loss [13]. In addition to sex and age, the population-specific patterns of mandible development have also been studied [14].

Regarding the collection of mandible information, the studies have traditionally relied on dry bone measurements. However, recent decades have seen the increasing use of imaging techniques, such as 3D optical scanner [11, 15], or computed tomography [8]. One of the most used systems is the panoramic dental imaging or orthopantomography (OPG), but this procedure has several drawbacks. Given its rotational acquisition process, the image projection leads not only to an information loss, but also to a potential deformation, which is especially noticeable in the horizontal direction [16]. However, it is still nondestructive, it captures the complete mandible in a single image, which is both faster and beneficial for the storage of data and the measuring process, and it is reportedly useful to measuring the mandible [17]. Indeed, the value of OPG images has been proved in a variety of dentistry tasks, including the diagnosis of several clinical conditions [18, 19], surgery management [20], or forensic procedures [21]. Although this imaging technique has been used for decades, the mandible detection methods based on automatic image processing algorithms are still very scarce [22, 23].

The current study presents a two-step pipeline to describe the shape of the mandible automatically on OPG images, to use this description for a variety of purposes. In the first step of the proposed pipeline, a deep convolutional neural network (CNN) is applied to automatically extract the mandible contour. In a second step, four different descriptors are employed to characterize the mandibular shape, namely a set of linear distances and angles, the centroid size, the mandible variations with respect to the mean shape, and a set of parameters given by a shape model.

## Materials and methods

**Fig. 1:**
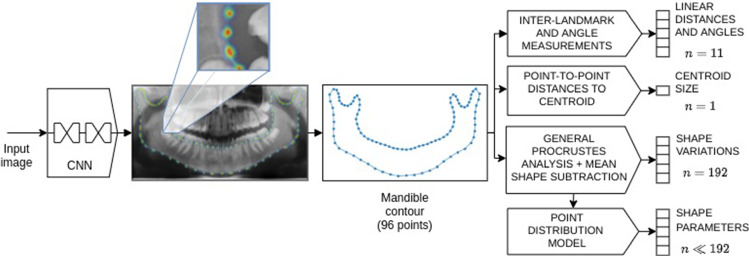
Process of describing the mandible shape from a new panoramic X-ray image. In a first step, the mandible contour composed of both landmarks and semilandmarks is obtained automatically with a CNN. In a second step, four descriptors are applied, including a set of linear distances and angles; the centroid size, the variations from the mean shape, and the shape parameters produced by a point distribution model.

The workflow employed in the present study is set out in Fig. 1. First, the contour of the mandible, given by a set of anatomical landmarks and the intermediate points—also known as semilandmarks—was obtained through an automatic landmark detection method based on a CNN. Second, four different descriptors were applied to the mandible contour, namely a set of 11 linear distances and angles, the centroid size, the shape variations with respect to the mean shape, and the shape parameters given by a point distribution model (PDM). Both steps are explained in detail in Sects. 2.2 and 2.3, respectively.

**Fig. 2:**
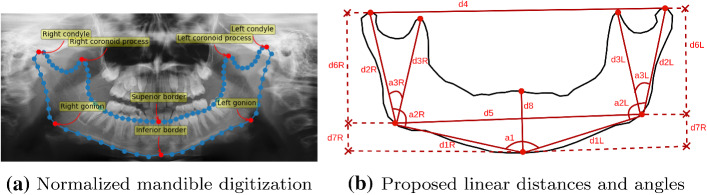
Mandible landmarks and measurements

### Data

This study uses an OPG dataset collected by the School of Medicine and Dentistry of the Universidade de Santiago de Compostela (Spain) with a direct digital panoramic unit (Orthophos Plus DS; Sirona USA, Charlotte, NC). All the images were 1,552 pixels high, with the width varying between 2,400 and 3,200 pixels. The dataset comprised 1,195 images of patients aged from five to 70, and the age and gender distributions were almost uniform.

The mandible contours were composed of eight anatomical landmarks, corresponding to the red points in Fig. 2, namely the right and left condyles (RC and LC), the right and left coronoid processes (RCP and LCP), the right and left gonions (RG and LG), and the superior and inferior borders (SB and IB). On top of that, 88 semilandmarks were placed along the mandible contour to fill the gap between anatomical landmarks. To minimize the potential errors associated with the semilandmarks’ placement [24], the annotators digitized them without a specific protocol regarding the position or the quantity. After that, they were automatically post-processed so there were a specific number of equally spaced semilandmarks between two consecutive anatomical landmarks (blue points in Fig. 2). Specifically, there were eight semilandmarks between the condyles and the gonions, eight between the gonions and the inferior border, 10 between the condyles and the coronoid processes, and 18 between the coronoid processes and the superior border. The normalized mandible shape, therefore, contained 96 points in every case. This manual digitization process was carried out through the Labelbox platform [25].

### Automatic digitization of the mandible contour

To make the mandible shape description method work in a fully automatic way, an automatic method to digitize the mandible landmarks and semilandmarks without the need for an operator is proposed. This was specifically approached as a heatmap regression problem. Therefore, a fully convolutional neural network was used to obtain one heatmap per contour point, i.e., 96. The target heatmaps were generated from a bivariate normal distribution, where the mean corresponded to the coordinates of the contour points, and the standard deviation was set to a fraction of the image width to ensure it works in the same way although the resolution of the image is changed.

The point coordinates were obtained from the estimated heatmaps using the soft-argmax function, which allows for sub-pixel precision; it is also differentiable, meaning that a network can be trained end-to-end. This was applied as follows: after estimating the heatmaps, each one was normalized so that its pixel values add up to 1. Then, the coordinates of every image pixel were multiplied by the heatmap value at those coordinates. The results were summed according to (1), where $\odot$ is the Hadamard product, *P* is the normalized heatmap, and *w* and *h* are the image width and height, respectively. This produced an approximation of the heatmap’s peak value.

1 $$\begin{aligned} \langle \widetilde{x_{max}}, \widetilde{y_{max}} \rangle = \sum _{x=1}^w \sum _{y=1}^h \Big [ \langle x,y \rangle \odot P_{x,y} \Big ]. \end{aligned}$$

After performing some experimentation with different state-of-the-art CNNs specifically designed for landmark localization, we selected the stacked hourglass network (SHN) [26]. This network involves the sequential application of a set of subnetworks representing a downsampling–upsampling architecture that relies significantly on residual connections to overcome the vanishing gradient problem. In the first stage, the network applies a set of convolution-pooling modules to output the probability map of each landmark. In successive stages, the subnetworks operate directly over the belief maps obtained in the previous stage, enabling the inter-landmark relationships to be modeled and, therefore, the results to be refined. The input image resolution was set to 256x512 pixels, and the SHN parameters were fixed to a depth of four and 64 initial filters. As an output, it produced 97 high-resolution outputs (one heatmap per contour point and one mandible mask).

**Table 1:** Description of the linear distances and angles.

**Code**	**Measurements**	**Description**
a1	Chin angle	Angle defined by the lines that join the gnathion and the mandibular angles
a2(L$\vert$R)	Mandibular angle	Angle formed by the lower margin of the body and the posterior margin of the ramus
a3(L$\vert$R)	Coronoid–condylar angle	Angle formed by the ramus and the imaginary line that connects the mandibular angle and the coronoid process
d1(L$\vert$R)	Diagonal length	Distance between the mandibular angle and the gnathion
d2(L$\vert$R)	Ramus length	Distance between the mandibular angle and the condyle
d3(L$\vert$R)	Coronoid–gonion	Distance between the gonion and the coronoid process
d4	Bicondylar breadth	Distance between the condyles
d5	Bigonial breadth	Distance between the gonions
d6(L$\vert$R)	Condyle-angle height	Vertical distance between the condyle and the mandibular angle
d7(L$\vert$R)	Angle-gnathion height	Vertical distance between the mandibular angle and the gnathion
d8	Chin height	Distance from interdental to gnathion

L$\vert$R: left and right sides

### Mandible description

The quantitative description of the mandible was performed using four different descriptors. First, the mandible contours given by the anatomical landmarks and semilandmarks were employed to calculate a set of linear distances and angles. Some of these measurements are widely used in forensics and other clinical procedures, such as the ramus length [27], the bigonial and bicondylar breadth [10]; and the mandibular angle [28]. Other additional measures have been proposed to further improve the mandible description. Overall, eight linear distances and three angles were considered, as set out in Fig. 2b and Table 1.

The second descriptor corresponded to the norm of the distances from each mandible contour point to the centroid and will be referred to as the centroid size [12]. To calculate the other two descriptors, the mandible contours were aligned through generalized Procrustes analysis (GPA) to provide optimal comparability. The mean shape ($\overline{\mathrm{X}}$) was calculated and subtracted from each aligned shape to obtain the vector of variations from the mean shape ($\Delta X$), which was used as the third descriptor. Finally, the fourth descriptor was computed using a point distribution model (PDM), which involved decomposing a shape into a mean shape and a linear combination of modes of variation [29].

We began with the shape variations, $\Delta X$, employing a singular value decomposition for each of them to transform $\Delta X$ into $U \Sigma V^{{\textsf {T}}}$, with: U being the matrix of the eigenvectors of $(\Delta \mathrm{X})(\Delta \mathrm{X})^{{\textsf {T}}}$; $\Sigma$ a diagonal matrix with the singular values; and $V^{{\textsf {T}}}$ the matrix of the eigenvectors of $(\Delta \mathrm{X})^{{\textsf {T}}}(\Delta \mathrm{X})$. The eigenvalues and eigenvectors were then extracted, the *i*-th eigenvalue giving the proportion of variance of the training shapes explained by the *i*-th eigenvector. As most of the shape variations could be represented with a reduced subset of modes of variation, the optimal number of modes required to explain a minimum proportion, *l*, of the total variance is computed from the eigenvalues.

To obtain the fourth descriptor, referred to as the shape parameters, the dataset was mapped to a *k*-dimensional space ($k\ll 2P$) using

2 $$\begin{aligned} (\Delta \mathrm{X})_k = (\Delta \mathrm{X}) V_k, \end{aligned}$$

where $V_k$ is a matrix composed of the first *k* columns of *V*. The original dataset, $\mathrm{X}$, was reconstructed via

3 $$\begin{aligned} \widetilde{\mathrm{X}} = \overline{\mathrm{X}} + (\Delta \mathrm{X})_k V_k^{{\textsf {T}}}. \end{aligned}$$

The PDM approach had three main benefits: 1. the dimensionality of the problem was reduced, while most of the shape variation was retained; 2. the low-dimensional shapes produced by $(\Delta \mathrm{X})_k$ were orthonormal to each other; and 3. it helped us to conduct graphical assessments of variations in the mandible’s shape.

### Comparative analysis

In this section, two different experiments were described, namely the validation of the automatic mandible digitization system, and the assessment of the predictive capabilities of the proposed mandible descriptors in the problem of sex and age estimation.

#### Mandible digitization

The first experiment comprised the comparison between automatic and manual mandible digitization methods. In this regard, the error produced by the CNN was compared to the interobserver error. To make this possible, a subset of 300 images from the dataset were annotated by a second expert. The results were compared by using the following metrics: the point-to-point error corresponding to the Euclidean distance between the real and estimated anatomical landmarks; the point-to-curve error (PT2CRV) corresponding to the minimum Euclidean distance between each estimated point (both landmarks and semilandmarks) to the real mandible contour, averaged over all the estimated points; the absolute error of the linear distances and angles; and the overlapping of the mandible masks through the Dice similarity coefficient (DSC). All the errors calculated through Euclidean distances were reported in mm by using the resolution information of the X-ray acquisition device (11.11 pixels/mm).

**Table 2:** Annotation errors of the 2nd observer and the prediction errors of the best-performing network (SHN), both of which are measured against the gold standard (1st observer). All the errors calculated are reported in mm

**Metric**		**Absolute error** ($\varvec{\mu }\pm \varvec{\sigma }$)	
		**Interobserver**	**Network**
Point-to-point absolute error (mm)	RG	4.73 ± 2.93	3.23 ± 2.59
	LG	3.85 ± 2.83	3.21 ± 2.31
	SB	1.20 ± 1.36	1.43 ± 1.26
	IB	1.58 ± 1.35	1.60 ± 1.52
	RC	1.08 ± 0.87	0.99 ± 0.75
	LC	1.44 ± 1.27	1.13 ± 0.92
	RCP	2.09 ± 2.28	1.40 ± 1.49
	LCP	2.35 ± 2.35	1.55 ± 1.65
Point-to-curve (mm)	PT2CRV	0.20 ± 0.09	0.21 ± 0.23
Angles absolute error (degrees)	a1 (chin angle)	2.57 ± 1.68	1.45 ± 1.42
	a2 (mandibular angle) ($^{(a)}$)	0.81 ± 0.71	0.81 ± 0.62
	a3 (coronoid–condylar angle) ($^{(a)}$)	1.95 ± 1.49	1.27 ± 1.09
Linear distances absolute error (mm)	d1 (diagonal length) ($^{(a)}$)	3.29 ± 2.24	2.24 ± 1.78
	d2 (ramus length) ($^{(a)}$)	3.69 ± 2.40	2.19 ± 1.85
	d3 (coronoid–gonion)	2.17 ± 1.83	1.39 ± 1.39
	d4 (bicondylar breadth)	1.37 ± 1.30	1.28 ± 1.09
	d5 (bigonial breadth)	4.60 ± 3.29	3.43 ± 1.60
	d6 (condyle-angle height) ($^{(a)}$)	3.50 ± 2.27	2.06 ± 1.79
	d7 (angle-gnathion height) ($^{(a)}$)	3.22 ± 2.13	1.91 ± 1.77
	d8 (chin height)	0.92 ± 0.89	0.70 ± 0.74
Mask overlapping	DSC	0.98 ± 0.01	0.99 ± 0.00

(a) Average on right and left sides

**Fig. 3:**
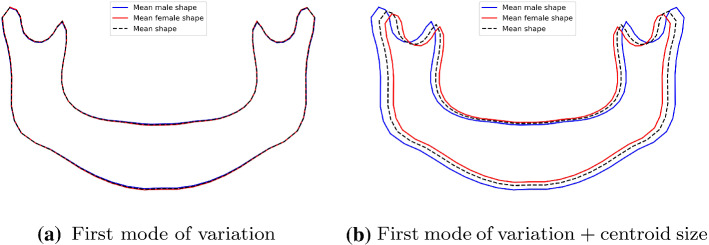
Mandible variations in subjects older than 18 regarding the sex

**Fig. 4:**
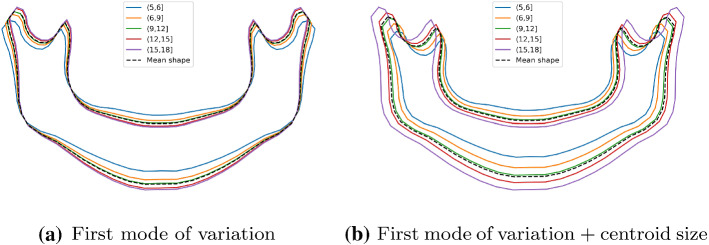
Mandible variations in subjects younger than 18 regarding the age

#### Sex and age estimation

In the second experiment, the proposed mandible description method was validated in a real problem representative of mandible change and widely studied in the literature: sex and chronological age estimation. In this regard, both the shape parameters and the centroid size were used to make a visual assessment of the mandible variations according to the sex and age of a subject.

Furthermore, predictive models were developed for sex and age estimation by using each of the proposed mandible descriptors as the independent variables. To avoid potential collinearity problems, especially with the linear distances and angles, ridge regression and classification models were used for age and sex estimation, respectively. To evaluate the robustness of the proposed automatic approach, the results obtained with the CNN-digitized mandible contour were compared with those obtained with a manual digitization process—referred to as the semiautomatic method.

The sex estimation performance was evaluated through the accuracy metric—the percentage of images correctly classified—and the F1. The latter is considered a more robust method for binary classification problems, and it is calculated independently for each class $C \in \{Male, Female\}$, as follows:

4 $$\begin{aligned} F1_C = \frac{TP}{TP + \frac{1}{2} (FP+FN)} \end{aligned}$$

where *TP* (true positives) is the number of images of the class *C* which are correctly classified, *FP* (false positives) is the number of images of the opposite class which are classified as the class *C* and *FN* (false negatives) is the number of images of the class *C* which are classified as the opposite class.

On the other hand, the age estimation performance was assessed through the absolute error between the real and estimated ages.

Furthermore, the best sex and age estimation models obtained in the previous step were compared to other methods proposed in the literature. The metrics in this comparison were those provided by the other researchers, namely the accuracy in the case of sex classification, and the standard error (SE), the coefficient of determination ($R^2$) and the *p* value associated with the F-test in the case of age regression.

As previously mentioned, sex and age estimations are more successful for specific age ranges. As a result, and to enable a reliable comparison with other methods, the sex estimation models were tested on subjects older than 18 and age estimation on those below that age.

## Results

In this section, the results concerning the experiments described in the previous sections are presented.

### Mandible digitization

As shown in Table 2, the greatest interobserver agreement on the issue of landmark digitization occurred for the condyles (1.08 and 1.44 for RC and LC, respectively), and the biggest differences were related to gonion localization (4.73 and 3.85 for RG and LG, respectively). Comparatively, the network yielded lower errors for every landmark other than the SB (1.20 vs. 1.43) and IB (1.58 vs. 1.60). The maximum difference was found for the RG, where the network reduced the degree of error by an average of 1.5mm. Concerning the linear distances and angles, the interobserver agreement in the angles was noticeably reduced by the network in the case of the chin (a1, 2.57 vs. 1.45) and coronoid–condylar (a3, 1.95 vs. 1.27) angles. The smallest interobserver error in the distance measurements was found for the chin height (d8, 0.92), while the greatest disagreement by far related to the bigonial breadth measurement (d5, 4.60). The neural network was also capable of reducing the differences between the observers and was especially noticeable for the diagonal length (d1, 3.29 vs. 2.24), ramus length (d2, 3.69 vs. 2.19), bigonial breadth (d5, 4.60 vs. 3.43), condyle-angle height (d6, 3.50 vs. 2.06), and angle-gnathion height (d7, 3.22 vs. 1.91), with a reduction of more than 1 mm for all of them. Overall, the overlapping of the mask of the mandible contour was slightly better with the mask estimated by the network (0.98 vs. 0.99).

### Sex and age estimation

For both the sex and age estimations, the first shape variation mode produced by the PDM was the most significant in relation to the classification/regression models. To visualize the main differences between the male and female mandibles, the mean male and female shapes were reconstructed using only the first mode ((3), with $k=1$). Furthermore, the effect of the mandible size was also assessed by scaling the mean reconstructed shapes with the mean male and female centroid sizes. Fig. 3a demonstrates that the mean adult male mandible shape is very similar to the female mandible shape. However, when the mean centroid size is included (Fig. 3b), the male mandible tends to be slightly bigger than that of the adult female subjects. The mean mandible shape is also reconstructed for the different age groups. As shown in Fig. 4a, the younger age group had more open rami, while the older age groups had a more pointy chin. When the size component is added, a clear mandible growing pattern can be seen (Fig. 4).

Table 3 compares the results of the semiautomatic and automatic methodologies, and is where it can be seen that the performance differences between them varied greatly depending on the mandible information used. When the linear distances and angles were employed in a fully automatic way, the accuracy increased by 2%. The classification method based on the centroid size yielded similar results both for the semiautomatic and the automatic approaches, with an accuracy value of about 0.750, while the performance for shape variations fell slightly with the automatic approach. The use of the shape parameters produced by the PDM led to better results in the automatic approach, with an improvement of 1.9% of accuracy and a more balanced F1 measure between males and females. Finally, the combination of the shape parameters and the centroid size produced the best results in every aspect. Specifically, the automatic approach outperformed the semiautomatic method by almost 2%, reaching an overall accuracy of 0.878. The F1 metric was also the highest, with values of 0.857 and 0.894 for males and females, respectively.

**Table 3:** Performance of the sex-classification method in those aged between 18 and 70.

**Predictor**	**Semiautomatic**			**Automatic**		
	**Acc**	$\mathbf{F1} _\mathbf{male }$	$\mathbf{F1} _\mathbf{female }$	**Acc**	$\mathbf{F1} _\mathbf{male }$	$\mathbf{F1} _\mathbf{female }$
Linear distances and angles	0.808	0.754	0.842	0.821	0.778	0.849
Centroid size	0.756	0.694	0.798	0.750	0.683	0.798
Shape variations	0.769	0.739	0.793	0.756	0.708	0.791
Shape parameters	0.731	0.691	0.761	0.750	0.748	0.769
Shape parameters + centroid size	0.859	0.831	0.879	0.878	0.857	0.894

RC: ridge classification; Acc: accuracy; Mean F1: F1 measure, averaged over both sexes

**Table 4:** Comparison of the sex-classification results in the literature (semiautomatic) and those of the best-performing automatic approach presented in this paper.

**Work**	**Age**	**Meas.**	**Method**	**N**	**Test**	**Acc** $^{(a)}$	**Acc ofthis work**$^{(a)}$ $^{(b)}$
Saini et al. [31]	23–65	DB (5)	DFA	116	–	0.802	0.881 (+ 7.9%)
Giles [32]	21–75	DB (9)	DFA	265	TT	0.850	0.871 (+ 2.1%)
Steyn and Işcan [10]	–	DB (5)	DFA	81	–	0.815	-$^{(c)}$
Dayal et al. [36]	25–69	DB (6)	DFA	60	CV	0.839	0.847 (+ 0.8%)
Pokhrel and Bhatnagar [9]	–	DB (4)	DFA	79	–	0.829	-$^{(c)}$
Abualhija et al. [21]	21–45	OPG (3)	LoR	50	TT	0.800	0.857 (+ 5.7%)
Franklin et al. [11]	18–70	3DS (38)	PDM+LoR	225	CV	0.831	0.878 (+ 4.7%)
Lin et al. [8]	21–70	3D CT (10)	DFA	240	LOO	0.879	0.871 (− 0.8%)
This work	18–70	OPG (96)	RC	935	TT	0.878	

(a) Shape parameters and centroid size were used, as they yielded the best results (Table 5)

(b) The accuracy was calculated for the same age range than original publications. The percentage differences were also reported

(c) The accuracy could not be calculated for the same age range, as the original work did not report this information

Meas.: Measurements. Meas. legend: DB: dry bone; 3DS: 3D scanner; CT: computed tomography. Method legend: DFA: discriminant function analysis; LoR: logistic regression; PDM: point distribution model; RC: ridge classification. N: sample size. Test approach legend: TT: train-test; CV: cross-validation; LOO: leave-one-out. Acc: accuracy

**Table 5:** Mean and standard deviation of the absolute error (in years) in the age estimation method for subjects aged between five and 17.

**Predictor**	**Absolute Error** ($\mu \pm \sigma$)	
	**Semiautomatic**	**Automatic**
Linear distances and angles	1.75 ± 1.24	1.80 ± 1.28
Centroid size	2.40 ± 1.83	2.38 ± 1.83
Shape variations	1.57 ± 1.17	1.79 ± 1.17
Shape parameters	1.82 ± 1.26	1.70 ± 1.09
Shape parameters + Centroid size	1.53 ± 1.26	1.57 ± 1.21

**Table 6:** Comparison of the best age estimation results of the automatic methodology and the semiautomatic results presented previously in the literature.

**Work**	**Age**	**Meas.**	**Method**	**N**	**SE**	R$^{2}$	**p**
Franklin and Cardini [37]	1–17	3DS (38)	LR	79	2.4	0.834	1$\times$10$^{-31}$
Franklin et al. [15]	1–17	3DS (38)	LR	79	2.1	0.880	1.8$\times$10$^{-37}$
			PDM+LR		2.4	0.827	1.8$\times$10$^{-27}$
This work	5–17	OPG (96)	RR($^{(a)}$)	260	2.0	0.804	0.00055

(a) Shape parameters and centroid size were used, as they yielded the best results (Table 5)

Meas. legend: 3DS: 3D scanner. Method legend: LR: linear regression; PDM: point distribution model; RR: ridge regression; N: sample size; SE: standard error (in years); R$^{2}$: coefficient of determination; p: *p* value of the F-test

The results produced by the automatic sex classifier were compared to the outcomes of the methods by other researchers reporting an accuracy greater than 0.8, as set out in Table 4. To enable a reliable comparison to be made, the findings are reported for the same age ranges used by these other authors. The proposed automatic method outperformed the other approaches in seven out of eight comparisons, with differences between −0.8% and +7.9%.

The age estimation results are presented similarly in Table 5. Each of the four descriptors yielded similar results when applying the semiautomatic or the fully automatic method. The main differences were obtained with the shape variations (1.57 and 1.79 for semiautomatic and automatic mode, respectively) and the shape parameters (with an improvement of 0.12 years in the error of the automatic mode). The best-performing descriptors were the shape variations and the shape parameters in the semiautomatic and fully automatic methods, respectively. When combining the shape parameters and the centroid size descriptors, the absolute error of both approaches was significantly enhanced (with improvements of 0.04 and 0.13 on average, respectively, with respect to the best performing single-descriptor model).

The age estimation methods were compared to those proposed by other authors with the same performance metrics, as set out in Table 6. Specifically, the performance of the proposed approach was reported for the subadult age range available in our dataset (5–17 years). Although the R$^{2}$ values were slightly worse (maximum of 0.880 vs. 0.804), our method outperformed these methods in terms of the SE (maximum of 2.4 vs. 2.0).

## Discussion and conclusions

This paper presents an automatic method for detecting and describing the mandibular contour. The mandible detection was carried out with the stacked hourglass network. This CNN produced, by a large margin, more confident detections than those of the experts for every anatomical landmark other than SB and IB and for every angle and linear measurement, as well as in the overlapping of the mandible mask.

To perform the quantitative description of the mandible, four different descriptors have been proposed. The combination of the shape parameters and the centroid size not only allowed us to summarize the shape and size information numerically, but to also produce comprehensive visualizations of the mandible variations between different populations, age cohorts, and sexes. In this regard, the first and main shape parameter given by the PDM represented a shape evolution in accordance with that reported in the clinical literature [14, 30]. This fact led us to confirm that the proposed approach is useful to assess the mandible shape changes both quantitatively and qualitatively.

Finally, all this shape information was used to compare mandible description for both a semiautomatic and a fully automatic method for the selected validation experiment of classifying sex and estimating chronological age. The two methods were then evaluated in five different scenarios: linear distances and angles; centroid size; shape variations; mandible shape parameters provided by the PDM; and mandible shape parameters together with centroid size. The semiautomatic method required an expert to annotate the mandible contour’s landmarks, which were then used to estimate both the sex and the age. The automatic method retrieved the mandible contour extracted by the CNN.

Concerning the sex-classification experiments, the top accuracy of the semiautomatic and automatic methods was achieved when combining the shape parameters and the centroid size. The F1 values of over 0.83 for both classes confirmed that the models were not biased toward a specific gender. The accuracy fell slightly when we used size-free descriptors alone, such as the shape variations and the shape parameters, or linear distances and angles. However, it is notable that the automatic method achieved a higher accuracy when relying on linear distances and angles. This is in line with the significant performance differences between the network and the observers when extracting these measurements.

Comparing the sex-classification performance with that of previous studies, the proposed methodology outperformed almost every other methodology except the approach in [8], which used 3D CT images. It is also notable that three out of the eight studies we analyzed did not describe any validation scheme [9, 10, 31], while one performed a train-test split on part of the dataset [32]. It should also, therefore, be noted that the data sample used by our team is composed of 935 images, making it the largest database used in an investigation of this kind.

Regarding the age estimation results, the absolute error of the proposed automatic method was between 1.57 and 2.38 years on average. Although the proportion of the explained variance given by R$^{2}$ was slightly lower than in the other methods, the proposed method performed better concerning the SE. This is especially remarkable, given that our study did not include subjects younger than five; if it had been done, the results may have been even better, due to the significant development that occurs in that age range.

Although the studies using CNN-based methods that employ an entire OPG image to conduct sex and age estimations performed better, they only serve the purpose for which they were developed [33–35]. On the other hand, the method we propose based on automatic mandible description performs well when estimating age and sex; it is also more versatile, as it can also be employed in other applications, such as in evaluating the mandible shape differences between populations, sexes, and age cohorts, and for disease diagnosing or surgery management.

In conclusion, the automatic method we describe in this paper is very reliable when extracting the mandible contour, with a dramatic improvement in the time it took to do so. Consequently, the methodology proposed, including the shape model, can be valuable in any field that requires a quantitative description of the mandible shape and a visual representation of its changes, such as clinical practice, surgery management, dental research, or legal medicine.

## Supplementary Information

Below is the link to the electronic supplementary material.

Supplementary material 1 (pdf 238 KB)

## Data Availability

Please contact the corresponding author for data requests.

## Code Availability Statement

Please contact the corresponding author to request the custom codes used in this study.
