# Supplementary material for: Automated description of the mandible shape by deep learning
Source: Int J Comput Assist Radiol Surg. 2021 Aug 27;16(12):2215–24. doi: 10.1007/s11548-021-02474-2 (PMC8616887; doi:10.1007/s11548-021-02474-2)
Supplement: Supplementary file 1 — Supplementary material 1 (pdf 238 KB) [file 11548_2021_2474_MOESM1_ESM.pdf]

Supplementary Material for "Automated Description of the Mandible Shape by Deep Learning"

Nicolás Vila-Blanco, Paulina Varas-Quintana, Ángela Aneiros-Ardao, Inmaculada Tomás, María J. Carreira

This supplementary material explains all the experiments performed to select the best architecture of CNN for an accurate digitization of the mandible contour landmarks.

1 Automatic localization of the mandible contour

The automatic extraction of the mandible contour landmarks was approached as a heatmap regression problem. Specifically, a Fully Convolutional Network (FCN) was used to obtain one heatmap per each contour point, i.e., 96. The target heatmaps were generated from a bivariate normal distribution, where the mean μ corresponded to the coordinates of the contour points and the standard deviation σ was set to a proportion of the image width to ensure it was agnostic of the input resolution at which the network functioned. Specifically, it was set empirically to $\sigma = I_w/85$, with I_w representing the width of the image.

The point coordinates were obtained from the estimated heatmaps using the soft-argmax function, which allows for sub-pixel precision; it is also differentiable, meaning that a network can be trained end-to-end. This was applied as follows: after estimating the heatmaps, each one was normalized so that its pixels totaled 1. Then, the coordinates of every image pixel were multiplied by the heatmap value at those coordinates. The results were totaled according to (1), where $\odot$ is the Hadamard product, P is the normalized heatmap, and w and h are the image width and height, respectively. This produced an approximation of the heatmap's peak value.

$$\langle \widetilde{x_{max}}, \widetilde{y_{max}} \rangle = \sum_{x=1}^{w} \sum_{y=1}^{h} \Big[\langle x, y \rangle \odot P_{x,y} \Big] \tag{1}$$

Four state-of-the-art FCNs specifically designed for landmark localization were compared: 1. the Convolutional Pose Machines (CPMs) [1]. These involve the sequential application of a set of subnetworks. In the first stage, the network applies a set of convolution-pooling modules to output the probability map of each landmark (both anatomical landmarks and semilandmarks). In successive stages, the subnetworks operate directly over the belief maps obtained in the previous stage, enabling the inter-landmark relationships to be modeled and, therefore, the results to be refined; 2. the Stacked Hourglass Network (SHN) [2]. This is a similar approach to [1], but in this case each subnetwork represents a downsampling-upsampling architecture and relies significantly on residual connections to overcome the vanishing gradient problem; 3. the Cascade Pyramid Network [3]. This architecture is composed of a Feature Pyramid Network (FPN) to detect the easiest landmarks, and a refinement network fed with the multi-resolution representations learned by the FPN to improve the detection of hard landmarks; and 4. the Deep High-Resolution Network (HRNet) [4], which consists of several branches working in

parallel at different resolutions. These branches are fused at intermediate points, providing rich multi-resolution representations.

Although it was not a required output, we added the mandible mask as an output to increase the mandible information provided to the networks and, as a consequence, improve their performance. The goal was for each network to produce 97 high-resolution outputs (one heatmap per each contour point and one mandible mask).

Three different losses were applied to train the networks: 1. a Binary Cross-Entropy Loss for the heatmap regression (2); 2. a Dice Loss for the mask prediction (3); and 3. a Root-Mean Squared Loss for the point localization (4), where Y is the ground truth element, $\hat{Y}$ is the predicted element, P is the number of points of the mandible contour (in this case, 96), w and h are the image dimensions, x and y are the point coordinates, and ϵ is a small number used to avoid a division by zero. These three losses were combined in a single function as the logarithm of the product (5).

$$\mathcal{L}_h(Y,\hat{Y}) = -\frac{1}{P\cdot w\cdot h}\sum_{p=1}^{P}\sum_{x=1}^{w}\sum_{y=1}^{h}\Bigg[\Big[Y_{p,x,y}\cdot\log(\hat{Y}_{p,x,y})\Big] + \Big[(1-Y_{p,x,y})\cdot\log(1-\hat{Y}_{p,x,y})\Big]\Bigg] \tag{2}$$

$$\mathcal{L}_m(Y,\hat{Y}) = 1-\frac{2\cdot(Y\odot\hat{Y})}{\sum Y+\sum\hat{Y}+\epsilon} \tag{3}$$

$$\mathcal{L}_p(Y,\hat{Y}) = \sqrt{\frac{1}{P\cdot w\cdot h}\sum_{p=1}^{P}\sum_{x=1}^{w}\sum_{y=1}^{h}\Big[Y_{x,y}-\hat{Y}_{x,y}^2\Big]} \tag{4}$$

$$\mathcal{L} = \log\left(\mathcal{L}_m\cdot\mathcal{L}_h\cdot\mathcal{L}_p\right) \tag{5}$$

2 Validation experiments

The mandible segmentation methods based on CNNs were compared in terms of the point-localization error. The input image resolution was set to 256x512 pixels, and we used different hyperparameter tuning for each network. The SHN was evaluated for different depths (four, five and six) and for different initial filters in each stage (16, 32 and 64). The number of stages was set to two. The CPM was tested with one and two refinement stages, and with 64, 128 and 256 initial convolutional filters for each stage. The CPN was evaluated with two different backbones, namely Resnet50 and Resnet101. Finally, the HRNet performance was assessed using different network sizes (C parameter in [4]), specifically 24, 48 and 96.

The performance of the four networks was evaluated through a set of metrics shown in Figure 1: the landmark-to-landmark error corresponded to the Euclidean distance between the real and estimated anatomical landmarks; the point-to-point error (PT2PT) was obtained via the Euclidean distance between the real and estimated contour points (both landmarks and semilandmarks); and the minimum

point-to-curve error (PT2CRV) corresponded to the minimum Euclidean distance between each estimated point (both landmarks and semilandmarks) and the real mandible contour, averaged over all the contour points. All the errors were calculated in mm by using the resolution information of the X-ray acquisition device (11.11 pixels/cm). The data were split into training (800 images), validation (200 images) and test (200 images) sets, and this division was retained in every experiment. The three subsets were equally distributed in terms of the age and gender of the subjects. The networks were trained with the Adadelta optimizer, and the batch size was set to four as a good compromise between the convergence speed and regularization effect. Image augmentation was applied to increase the dataset's variability, including the horizontal flip, translation, rotation, and contrast and brightness disturbance [5].

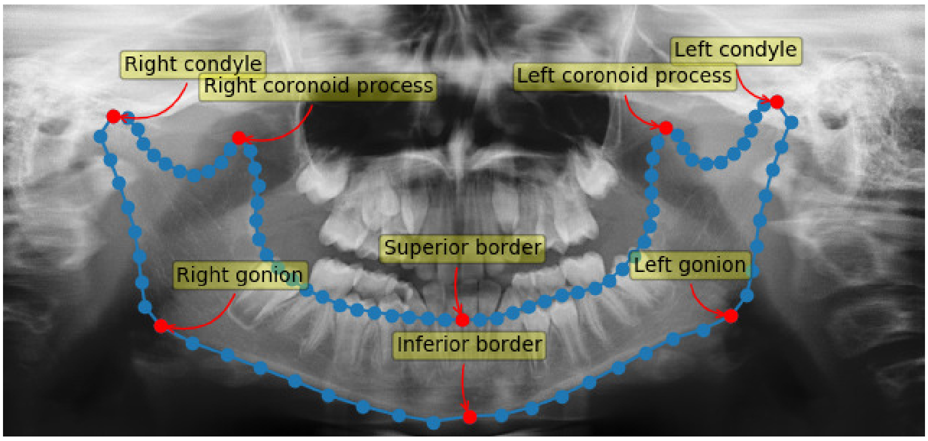

Fig. 1: Normalized annotation, with a fixed number of points between each pair of consecutive landmarks.

3 Results of CNN comparison

The performances of the four tested architectures are set out in Table 1. First, to identify the best-performing setup in terms of the point-to-point error, the different configurations of each network were trained without including the mandible mask as an additional output. Although the differences were almost negligible: the SHN performed better with a depth of four and 64 initial filters; the CPM had better results with one refinement stage and 256 initial filters; the CPN functioned best with Resnet50 as the backbone network; and the optimal performance of the HRNet was achieved with a size of 48.

Without including the mandible mask as an additional network target, the gonions were the worst localized anatomical landmarks, with mean errors between 3.1 (SHN) and 3.5 (CPM) mm for the RG and between 3.4 (HRNet) and 3.5 (CPN) mm for the LG. The SB errors ranged from 1.4 (HRNet) to 2.0 (CPN) mm, and

the IB errors from 1.7 (HRNet) to 1.9 (SHN and CPM) mm. The lowest errors were yielded for the condyles, ranging from 1.2 (SHN) to 1.4 (CPN) mm for the RC, and between 1.3 (SHN) and 1.5 (CPM) mm for the LC. Finally, the coronoid processes were best estimated by the SHN, with mean errors of 1.7 and 1.8 mm for the RCP and LCP, respectively, while the worst were localized by the CPM, yielding a mean error of 2.2 and 2.3 mm. The lowest point-to-point (PT2PT) and point-to-curve (PT2CRV) errors were achieved by the HRNet, at 1.8 and 0.2 mm, respectively.

These network setups were retrained from scratch with the inclusion of the mandible mask. The impact of this addition differed depending on the network: 1. in the SHN, the performance increased for every metric other than the RG error. The improvement on landmark localization varied between 0 mm on the SB and 0.2 mm on both the LG and the RCP. The PT2PT errors also decreased, by an average of 0.1 mm, respectively; 2. the CPM improved the results noticeably in the RG, IB, LC, and LCP, but performed worse in the SB. The PT2PT error remained unchanged; 3. there was a noticeable improvement in the CPN with respect to the localization of both gonions and the SB and IB, but the localization accuracy fell for the other landmarks. The PT2PT and PT2CRV errors followed the same pattern as the CPM, with a minor degradation of 0.1 mm for the latter; and 4. the HRNet improved for all the landmarks other than the LCP. The mandible contour fitting was also improved, with a reduction in the PT2PT errors.

Overall, the SHN with the addition of the mask outperformed the rest of the approaches tested, with the best results in the PT2PT and PT2CRV errors; it also produced the smallest errors in six out of eight anatomical landmarks. It was therefore used in the experiments described in what follows.

4 Discussion and Conclusions

The mandible detection was carried out with a CNN. In particular, four different state-of-the-art CNN architectures were compared. The Stacked Hourglass Network with 64 initial filters and a depth of four produced the best estimations in almost every anatomical landmark that were tested. The overall contour fitting was also the best, with the lowest point-to-point and point-to-curve errors: an average of 1.7 and 0.2 mm, respectively. It is remarkable that the addition of the mandible mask to the network output contributed to increasing the contour-detection performance.

References

1. S-E Wei, V Ramakrishna, T Kanade, and Y Sheikh. Convolutional pose machines. In *Proc CVPR IEEE*, pages 4724–4732, 2016.
2. A Newell, K Yang, and J Deng. Stacked hourglass networks for human pose estimation. In *Computer Vision – ECCV 2016*, pages 483–499. Springer, 2016.
3. Y Chen, Z Wang, Y Peng, Z Zhang, G Yu, and J Sun. Cascaded pyramid network for multi-person pose estimation. In *Proc CVPR IEEE*, pages 7103–7112, 2018.
4. K Sun, B Xiao, D Liu, and J Wang. Deep high-resolution representation learning for human pose estimation. In *Proc CVPR IEEE*, pages 5693–5703, 2019.
5. N Vila-Blanco, MJ Carreira, P Varas-Quintana, C Balsa-Castro, and I Tomás. Deep neural networks for chronological age estimation from opg images. *IEEE Trans Med Imaging*, 39(7):2374–2384, 2020.

Table 1: Point detection absolute error ($\mu \pm \sigma$) of the best configurations for each CNN model, with and without the mandible mask as an additional target. RG: right gonion; LG: left gonion; SB: superior border; IB: inferior border; RC: right condyle; LC: left condyle; RCP: right coronoid process; LCP: left coronoid process; PT2PT: point-to-point errors measured over all mandible points; and PT2CRV: point-to-curve errors, also measured over all mandible points. [(a)]

Model	RG*	LG*	SB*	IB*	RC*	LC*	RCP*	LCP*	PT2PT*	PT2CRV*
**SHN** [2]	**3.1** ± **2.5**	3.5 ± 2.5	1.5 ± 1.3	1.9 ± 1.6	1.2 ± 0.8	1.3 ± 0.9	1.7 ± 1.9	1.8 ± 2.0	1.8 ± 1.6	0.3 ± 0.3
**SHN+Mask**	3.2 ± 2.6	**3.2** ± **2.3**	1.4 ± 1.3	**1.6** ± **1.5**	**1.0** ± **0.7**	**1.1** ± **0.9**	**1.4** ± **1.5**	**1.5** ± **1.6**	**1.7** ± **1.5**	**0.2** ± **0.2**
**CPM** [1]	3.5 ± 2.7	3.5 ± 2.4	1.5 ± 1.2	1.9 ± 1.6	1.4 ± 0.9	1.5 ± 1.2	2.2 ± 2.0	2.3 ± 2.1	1.9 ± 1.5	0.3 ± 0.3
**CPM+Mask**	3.3 ± 2.5	3.5 ± 2.4	1.7 ± 1.5	1.8 ± 1.6	1.4 ± 0.9	1.4 ± 0.9	2.2 ± 1.9	2.1 ± 1.9	1.9 ± 1.5	0.3 ± 0.3
**CPN** [3]	3.4 ± 2.8	3.5 ± 2.6	2.0 ± 1.3	1.8 ± 1.5	1.4 ± 1.0	1.4 ± 1.0	2.0 ± 1.8	2.2 ± 1.9	1.9 ± 1.6	0.2 ± 0.3
**CPN+Mask**	3.3 ± 2.5	3.3 ± 2.2	1.4 ± 1.2	1.7 ± 1.5	1.5 ± 0.9	1.6 ± 1.1	2.0 ± 1.7	2.3 ± 1.9	1.9 ± 1.5	0.3 ± 0.3
**HRNet** [4]	3.3 ± 2.6	3.4 ± 2.5	1.4 ± 1.3	1.7 ± 1.7	1.3 ± 0.9	1.4 ± 1.0	1.9 ± 1.7	2.0 ± 2.0	1.8 ± 1.5	0.2 ± 0.3
**HRNet+Mask**	3.2 ± 2.7	3.2 ± 2.3	**1.4** ± **1.2**	1.7 ± 1.6	1.2 ± 0.9	1.4 ± 1.0	1.7 ± 1.8	2.1 ± 2.0	1.7 ± 1.5	0.2 ± 0.2

(a) Metrics given in mm by using the resolution information of the X-ray acquisition device (11.11 pixels/mm).
